# When Medicine Gets Irritating: Interactional and Structural Triggers of Uncertainty in Family Medicine Residency Training

**DOI:** 10.1111/jep.70607

**Published:** 2026-09-19

**Authors:** Konrad Hierasimowicz, Laura Purkl, Norbert Donner‐Banzhoff

**Affiliations:** ^1^ Department of Family Medicine University of Marburg Marburg Germany

## Abstract

**Background:**

Uncertainty is part of everyday medical practice, especially in family medicine. Former studies have focused on how clinicians manage uncertainty, but its emotional experience has received less attention. This study looks at irritation as a distinct affective entry point through which family medicine residents first perceive disruptions in clinical, interactional or organisational issues.

**Methods:**

Between 2019 and 2021, we spoke with 15 residents in Hesse, Germany. In semi‐structured interviews, they described situations where they felt uncertain, including the context, their emotions, and what they did next. We transcribed, pseudonymised and analysed the interviews using Kuckartz's qualitative content analysis.

**Results:**

In many cases irritation was the first emotional reaction when something in the consultation didn't feel right. We identified three common triggers: (1) a mismatch between intuition and medical findings, (2) irritation during patient interactions and (3) irritation caused by structural issues. The last two were our main focus, as they disrupt usual ways of working rather than pointing to hidden diagnoses. These moments of irritation could be confusing at first, but in some cases, they led residents to dig deeper into what shaped the patient's perspective, to ask colleagues for advice or pay more attention to systemic and organisational issues.

**Conclusion:**

Irritation acts as a harbinger of uncertainty by embodying those small breaks in routines and expectations from which uncertainty first arises. At first, it might throw residents off balance, but if they take the time to reflect on it, irritation can help them see things differently, stay attentive and improve their clinical skills. Residency programmes should give residents room to talk about these experiences, helping them handle uncertainty more effectively in their daily work.

## 1 Introduction

Uncertainty is an inherent aspect of medical practice, especially in family medicine, and has long been discussed in both medical and sociological literature. As Fox [1] showed, medical uncertainty does not decrease with scientific progress but changes its form and location. It may be placed in the world—in the complexity of illnesses, gaps in evidence or ambiguous outcomes—or in the self, as doubt, hesitation or emotional discomfort. Earlier literature often treated uncertainty as a deficit or failure of knowledge, as in Beresford's [2] work. More recent studies have examined how physicians deal with it productively. Based on qualitative interviews, Han et al. [3] developed a taxonomy of strategies for handling uncertainty, ranging from information‐oriented to relationship‐oriented approaches. In a more normative turn, Buetow [4] suggested that uncertainty can even be seen as a virtue, promoting creative reasoning, supporting safety and hope and protecting against excess. Together, these perspectives have brought attention to the emotional and relational dimensions of uncertainty, yet they have rarely examined how physicians themselves identify uncertainty in the flow of everyday practice.

Beyond the question of whether uncertainty is a deficit or a gain, other scholars have underlined its enacted and contextual dimensions. Timmermans and Angell [5] distinguished epistemic from practical forms of uncertainty, showing that medical doubt is not only a cognitive matter but also performed in social and organisational settings. This turn towards the enacted dimension provides an important bridge to our own focus on affective entry points.

In our earlier paper, we distinguished three types of uncertainty—biomedical, interpersonal and psychosocial—and examined their affective dimensions [6]. That typology focused on *content‐related sources* of uncertainty and coping strategies of residents. In the present article we shift our attention to the *affective entry points* of uncertainty as described in interviews with residents.

As Farnan et al. [7] noted, the experience and management of uncertainty is especially formative during residency. Unlike Buetow's normative argument, we focus here on the empirical level: how residents describe moments of irritation and what dynamics of action emerge. To categorise this contribution, it is useful to note that much research has addressed intuition and ‘gut feeling’. Stolper et al. [8] established gut feeling as a diagnostic track complementing analytical reasoning. Subsequent work by C.F. Stolper and colleagues showed that gut feelings are explicitly discussed in tutorial dialogues between GP trainees and supervisors [9]. Surveys in Denmark and Spain confirmed the relevance of gut feelings in practice and their association with empathy and a ‘sense of alarm’ [10, 11]. These affective signals differ from what have been described as cognitive ‘discrepancy heuristics’ (‘Diskrepanz‐Heuristiken’) [12] in which physicians consciously register a deviation from what they regard as normal or expected and use this perception as a basis for diagnostic reasoning. While discrepancy heuristics rely on reflective pattern recognition and comparative judgement, affective markers such as ‘gut feeling’ or irritation operate at a pre‐reflective level: they are felt before they are named, and they alert the physician to uncertainty even before it can be analytically framed. Taken together, this literature shows that gut feeling is a common affective marker of uncertainty.

By contrast, physicians' irritation as an affective marker has barely been investigated. Related insights appear in sociology and anthropology [13, 14, 15, 16, 17]. Balint [18] already described physicians' irritation and emotional discomfort as meaningful, though largely implicit, signals for reflective practice. Yet none has systematically conceptualised irritation in family medicine. The present article therefore extends our earlier analysis by examining how irritation emerges in family medicine residents' narratives and how it shapes the dynamics of uncertainty. Our analysis focuses not only on where uncertainty originates, but also on how it is *affectively marked and transformed* in practice.

We conceptualise irritation as an affective response when physicians' expectations, routines or communicative frames are disrupted.

Based on Goffman's concept of the ‘interaction order’ [19], Heritage [20] has shown how communication routines transferred from everyday talk into medical consultations can become dysfunctional, for instance in the presentation of problems, in the design of medical questions, or in negotiations over treatment recommendations. Such transfers of conversational norms into the clinical setting may produce nuanced disruptions that are not explicitly perceived as ‘uncertainty’, but are often embodied as discomfort or tension. Rather than a strictly cognitive awareness of uncertainty, these affective responses point to moments when the taken‐for‐granted flow of interaction loses its smoothness. They correspond with research on nonverbal communication in medicine, which shows how posture, facial expression and touch structure mutual understanding well before words are spoken [21, 22, 23, 24]. From this perspective, irritation can be understood as a physical and emotional registration of disruption—one that may precede, accompany or follow interpretation processes. In our study, we build on these insights to examine how family medicine residents describe such embodied disturbances in their narratives of uncertainty. In this sense, irritation does not simply cause a brief discomfort, but initiates a situational disturbance that shakes up familiar procedures and prompts physicians to reconsider meanings, diagnostic conclusions and professional boundaries. Our analysis focuses on how such disturbances make uncertainty visible and open pathways for working through it.

Yet, uncertainty in clinical practice does not always manifest through sudden disruptions: it may also evolve gradually—for instance from missing information, limited prior knowledge or the burden of having to decide alone—and is often accompanied less by acute reactions than by more subtle forms of emotional discomfort. Previous studies have scrupulously examined the biomedical dimension of such gradually emerging uncertainty, particularly the discrepancy between gut feeling and analytical reasoning [11, 25, 26, 27, 28]. Beyond the biomedical dimensions of uncertainty, we shift attention to the less theorised domains in which uncertainty is affectively marked: the interactional and structural conditions of practice. These perspectives guide our analysis of how irritation becomes visible as an affective entry point into professional reflection and organisational awareness.

Consequently, this paper addresses a blind spot in existing literature. Prior studies documented how gut feelings oscillate between alarm and reassurance, are based on experience, and are legitimised through guidelines or evidence. What has received far less attention are the ways in which irritation emerges from interactional breakdowns or from structural constraints such as time pressure, organisational demands or conflicting institutional logics. By foregrounding these intersubjective and systemic dimensions, our study extends uncertainty research beyond the intuition‐evidence dichotomy.

Against this background, our research question is: How do family medicine residents narrate their experience of irritation in moments that trigger uncertainty, and in what ways do interactional and structural disruptions unfold from initial paralysis into action dynamics? Furthermore, what implications do these processes have for the design of postgraduate training in family medicine?

## 2 Methods

In our work we conducted semi‐structured interviews with 15 family medicine residents between 2019 and 2021. Recruitment took place through seminars offered by the Hesse Family Medicine Training College at the Competence Center for Advanced Training in Family Medicine (Kompetenzzentrum Weiterbildung Hessen). Inclusion criteria were at least 3 months of experience in a family doctor's office and willingness to talk about personal experiences with uncertainty in the medical profession. Overall, the participants reported around 40 case examples. From these, we purposively selected seven in which interactional or structural irritation was articulated with particular clarity (intensity sampling). This selection foregrounds the phenomenon but over‐represents cases with a recognisable trajectory; less clearly resolved or unreflected episodes are correspondingly underrepresented (see Limitations).

Due to COVID‐19 restrictions, 14 of the 15 interviews were conducted remotely—11 by telephone and 3 by videoconference—while 1 took place in person. Sessions lasted between 24 and 72 min (mean 46 min). In advance of the interview, participants received a reflection guide to prepare two to four cases. This guide functioned as a memory aid and was not included in the analysis. During the interview, participants reconstructed these cases in detail and follow‐up questions explored the context, emotions, interactions, decisions and lessons learned. The design was reviewed and approved by the institutional review board of the University of Marburg. All interviews were recorded, transcribed and pseudonymised.

The approach followed Kuckartz's [29] method for qualitative content analysis. First, transcripts were openly and inductively coded in data analysis software to identify central patterns of affective markers of uncertainty. A coding system was then developed and iteratively refined in the research team. Discrepancies in coding were discussed to maintain consistency and to integrate different disciplinary perspectives.

AI‐based language tools (GPT‐5.1 and DeepL) were used for translational issues and linguistic refinement of the manuscript; they were not used for coding or analysis.

Ethical approval and informed consent are described in the Ethics Statement.

## 3 Results

The interviews show that residents frequently frame uncertainty in their narratives through affective markers. Such markers can in principle arise in biomedical as well as interpersonal or psychosocial constellations. In our material, three recurrent sources of irritation could be distinguished:

1. irritation caused by discrepancy between gut feeling and objective findings,
2. irritation in dealing with patients or relatives, which is typically perceived as a disruption of interactional order and
3. irritation arising from structural conditions such as language barriers, time pressure or organisational constraints.

Since the first form has already been widely described in previous research on gut feeling, our empirical analysis concentrates on the latter two.

### 3.1 Interactional Irritation

#### 3.1.1 Vague Discomfort and Enduring Doubt

##### 3.1.1.1 ‘The Dizziness, the Dizziness’: When Recurrent Complaints Signal Psychosocial Strain (Interview 12, Case 2: ‘Recurring Dizziness’)

A 73‐year‐old woman, long familiar to the doctor's office, consulted with striking frequency—initially for fluctuating hypertension, later for recurrent dizziness. She presented even small deviations in her self‐measured readings as emergencies and often arrived unannounced. *When her file came in once more, I thought: not her again*, the resident admitted, describing how repeated consultations led to frustration and the sense of dealing only with *trivialities*. Similar to the ‘heart‐sink’ phenomenon described in general practice [30], such affective responses signalled the physician's awareness of a recurring, relationally complex situation.

This attitude changed after a repeated referral to an ENT specialist as part of ongoing diagnostic clarification. On this occasion, the specialist identified vestibular neuritis. Suddenly irritation gave way to self‐doubt: *Maybe I was already biased and overlooked the possibility of an organic cause?* The dizziness apparently had a recognisable medical cause. However, even after appropriate treatment, the patient continued to experience similar symptoms. Over time, it became increasingly clear to the resident that her symptoms were worsened by depressive moods, family conflicts, and the strain of maintaining household and care responsibilities at the age of 73.

In this way, the case implied a dual causality: an acute organic disorder entangled with persistent psychosocial distress. Crucially, what began as exhausting repetitiveness gradually opened the resident's perspective. Irritation, initially paralysing, led to a broader and more empathetic understanding of the patient's situation—linking biomedical vigilance with awareness of psychosocial burden.

##### 3.1.1.2 Sharing the Burden: From Individual Failure to Structural Reality (Interview 7, Case 1: ‘Endless Chest Pain’)

In this case the resident recalled that irritation often set already in the moment she read the patient's name on the list: a woman over 90, also long familiar to the doctor's office. She appeared again and again in the overflow consultation with acute chest or abdominal pain. Despite repeated referrals and hospital examinations, no organic cause could be found. But her advanced age and history of heart disease and cancer worried the doctor: *Even when I am convinced it is psychosomatic, there is always this fear that one day it won't be.*

To deal with this tension, she relied on careful examinations and minimal interventions, but also sought conversation with the patient's daughter. When the daughter responded with calm understanding and acknowledged that at her mother's age a serious illness could one day go unnoticed because not every episode could be scrupulously investigated, the resident felt at least a little relieved. She no longer viewed uncertainty as mere individual failure, but reframed it as a systemic reality of care within limited temporal and diagnostic capacities. Nevertheless, the repeated interruptions at the overflow consultation and the impossibility of providing lasting relief for the patient repeatedly caused irritation—short waves of helplessness that kept recurring as soon as the limits of medical intervention became manifest.

#### 3.1.2 Breakdown of Routines in Chaotic Encounters

##### 3.1.2.1 When Interactional Dynamics Override Clinical Reasoning (Interview 13, Case 1: ‘Home Chaos’)

In the narrated case we describe here, a home visit took place to a previously unknown patient with chest pressure and presyncope. The home environment was described by the resident as highly unstructured and turbulent, and the social situation as well as the limited diagnostic possibilities made the assessment difficult. The patient cared for her sick husband and disabled child, and categorically refused hospitalisation despite acute symptoms. *I wasn't certain if it was a psychological reaction or a physical problem—and I had no diagnostic certainty*, the young physician remembered. Without an ECG, distracted by the trouble at home, and confronted with the patient's refusal, the usual routines broke down: neither clear history nor standard diagnostics were possible. Instead, the resident relied on documentation, consultation with his supervisor, and close follow‐up. Only later did an elevated troponin level confirm a mild heart attack.

The irritation came not only from ambiguous symptoms, but also from the breakdown of routines under social and structural pressure. The doctor realised that uncertainties could not be eliminated just through biomedical methods, but required communicative and organisational management. Sharing responsibility and openly acknowledging uncertainty became formative: irritation exposed the weakness of routine practice, but resulted in an understanding of how collegial support and exchange of experiences can strengthen professional action.

#### 3.1.3 Overwhelming and Threatening Encounters

##### 3.1.3.1 When Manipulative Turbulence Derails the Consultation (Interview 4, Case 1: ‘Drug Demand’)

The resident described this encounter as her *most uncertain case* in her training period so far. A patient presented with a cough and, surprisingly, asked urgently for dihydrocodeine. What began as a standard enquiry quickly turned into an incoherent and emotionally charged story about drug use, suicidal behaviour and trauma therapy. The resident became increasingly absorbed in the patient's recounting and struggled to keep her usual clinical professional distance. She was not only overwhelmed by the disorienting changes of topic and tone, but also confused by a modest role reversal: The patient sensed her uncertainty and tried to manipulate her by saying, *You're trained not to let this bother you. Don't worry.*

In that moment, the usual asymmetry of the consultation inverted. The patient claimed interpretive authority over the physician's affect, making the physician's professional composure an object of control. What followed was a quiet crossing of boundaries: under pressure, the resident broke with her routine and prescribed the medication against her own convictions. Only later, in collegial discussion, she could share the burden and reframe the episode as a lesson to involve colleagues earlier and not to bear such emotional tension alone. The case illustrates how affective overload can destabilise professional boundaries and role asymmetry, but can also be a driver for learning through reflection.

##### 3.1.3.2 Breakdown of the Interaction Order Under Threat (Interview 9, Case 2: ‘Aggressive Husband’)

What began as a routine appointment turned into one of the most disturbing experiences of this resident's training. When the resident was alone in the office in the morning, she was confronted by the agitated husband of a patient, who quickly became aggressive, shouting, leaning over her and blocking her way. She remembered: *I suddenly realised that I was alone with two assistants – there was no one else in the practice. It felt really threatening.* At that moment, medical care was interrupted. The doctor could not reach the patient, who sat there in despair and urged her husband to remain calm, while the consultation turned into fear and paralysis.

After both had left, the resident documented the incident, sought support from colleagues, and was reassured by her supervisor, who later confronted the man about his behaviour, whereupon she received an apology from him. Importantly, the incident led to lasting adjustments: the team got an alert function in the electronic system, and the resident adopted strategies such as briefly leaving the room to de‐escalate the situation and return more composed. In retrospect, she realised that both she and the patient's husband had, of course, been driven by concern for the patient, but had *completely talked past each other*.

This case shows how irritation in the form of an acute threat initially prevents medical measures, but through collegial support and organisational adjustment can become a stimulus for learning about de‐escalation and structural protection.

### 3.2 Structural Irritation

#### 3.2.1 Lost in Translation: Irritation Under Language Barriers (Interview 11, Case 1: ‘Sick Boy’)

A 2‐year‐old boy with fever and breathing difficulties was brought in by his father, who spoke little German. The resident suspected spastic bronchitis, prescribed inhalation therapy, and emphasised that the child should be seen by a doctor again if warning signs appeared. Her irritation grew when the father suddenly demanded a high dose of prednisolone: *Oh wow, that's quite a lot for such a small child*. She refused the request, and at the same time she feared that he might not understand the risks or recognise a deterioration in the child's condition. The case continued to occupy her thoughts even after work, so she eventually called a colleague at home. This colleague knew the family and reassured her that they were well embedded in local networks and would seek help if needed. In retrospect, the resident remarked that she should have asked the child's father to repeat what he had understood. What began as irritation over a dangerous request under fragile communication conditions transformed through collegial exchange and reflection on communication into a moment of learning.

#### 3.2.2 When Life Falls Apart in the Consultation Room (Interview 10, Case 1: ‘Thrown Out’)

Our last case involves a woman in her late thirties who repeatedly presents herself at the practice and is suffering from increasing psychosocial stress. Her problems stem, among other things, from the separation from her former partner and a dispute with the new one, which threatens to end in her being kicked out of his apartment. She had postponed a recommended psychotherapy, convinced she did not need it. The situation escalated when she came after being expelled out of her current partner's home, uncertain where to go and worried about her pets. The resident remembered: *The uncertainty was greatest when she said her partner had kicked her out. I had no solution for this acute situation*. The irritation here did not stem from diagnostic doubts, but from the confrontation with a crisis outside the professional framework of family medicine. Supportive talks and short‐term follow‐up care were possible, but the interviewee pointed out that such encounters often had to be handled more *by gut feeling and based on previous experiences* than with medical expertise. Structural constraints, including limited time, long waits for psychotherapy and patients unwilling or unable to seek it, amplified this helplessness.

Looking back, the resident highlighted two important insights: that she cannot solve everything within her professional capabilities, for example when patients refuse psychotherapy, but also that continuous availability and short‐term follow‐up can offer important support. She emphasised the importance of reflection among colleagues, whether in Balint groups or in case discussions, as a way of sharing responsibility. In this sense, the irritation proved instructive: it revealed the systemic limits of her role, but also highlighted the continuity of relationships and collegial exchange as resources for dealing with such cases.

An overview of all cases, including their sources of irritation, the resulting transformations and learning potentials, is summarised in Table 1. The analytical dimensions derived from these case trajectories are summarised in Table 2, which distils the findings into a general framework.

**Table 1:** Case summaries highlighting irritation, transformation and professional learning.

Interview, case	Resident characteristics (gender, age, training year)	Source of irritation	Transformation	Learning potential
12,2: ‘Recurring Dizziness’	Female, 35, 2nd year	Repetitive, seemingly trivial complaints	Shift from annoyance to recognition of psychosocial strain and ‘dual causality’	Patience; openness to psychosocial dimensions
7,1: ‘Endless Chest Pain’	Female, 37, 2nd year	Frequent psychosomatic‐appearing consultations	Insight that uncertainty is structural, not personal failure; relief via daughter's perspective	Normalisation of uncertainty; sharing responsibility
13,1: ‘Home Chaos’	Male, 31, 1st year	Collapse of diagnostic routines, no ECG available	Learning importance of documentation, supervision, shared responsibility	Acceptance of personal limits; organisational competence
4,1: ‘Drug Demand’	Female, 31, 1st year	Manipulation and loss of asymmetry	Recognition of need for boundaries and early collegial support	Self‐protection; teamwork; reflecting on power dynamics
9,2: ‘Aggressive Husband’	Female, 39, 1st year	Threat, loss of control	Organisational measures (alert system, de‐escalation)	Prevention; structural awareness; need for team support
11,1: ‘Sick Boy’	Female, 34, 1st year	Unsafe treatment request under poor communication	Collegial consultation reveals social context; reflection on communication strategies	Use of ‘teach back’; attention to family networks
10,1: ‘Thrown Out’	Female, 44, 1st year	Helplessness facing non‐medical problems	Realisation of value of continuity, listening, availability	Accepting limits of role; relationship continuity as resource

**Table 2:** Generalised framework of irritation sources and trajectories (derived from Table 1).

Source of irritation	Routine disrupted	Attention shift	Typical practices	Potential outcomes	Learning potential
Interactional irritation	Interactional order and relational routines disrupted (conflict, repetition, aggression)	From clinical task → to psychosocial context and power dynamics	Boundary‐setting, deeper listening, de‐escalation, collegial reflection	Reframing of patient situation; if unresolved, risk of fatigue, avoidance, moral distress	Enhanced capacity to integrate psychosocial dimensions; reflection on professional boundaries
Structural irritation	Organisational routines destabilised (time, language, documentation, chaotic home)	From individual case → to systemic or contextual conditions	Improvisation, network use, consultation with colleagues, referral	Recognition of limits of role; if unreflected, lingering helplessness, disengagement	Awareness of structural constraints; capacity to mobilise systemic resources

## 4 Synthesis

Our analysis of the case reports revealed two recurring developmental pathways—either a deeper understanding of the patients by the resident physicians or the uncovering of structural limitations—which manifested themselves in four analytical dimensions: temporal persistence, ethical considerations, agency and power, and organisational conditions. Each of these four dimensions is examined in more detail in the following subsections.

### 4.1 Temporal Persistence of Uncertainty

Irritation, as described in the accounts of the residents, was often the initial and emotional signal that something had gone wrong in the consultation. It did not represent the uncertainty itself, but rather pointed it out—it alerted the residents to a disturbance before its cause could be analytically framed. Interactional and structural irritations often overwhelmed the residents at first, but later reappeared as activators of further reflection or a deeper understanding (e.g. Interview 12, Case 2). In this way, uncertainty unfolded over time, extended beyond the immediate encounter and contributed to professional learning.

### 4.2 Risk and Ethical Balancing

In the interviews for the study, residents described dilemmas where the irritation marked the moment when they recognised a potential risk due to limited time and resources. In such situations, hesitation could lead to both the danger of medical underservice and the loss of trust (e.g. Interview 7, Case 1; Interview 11, Case 1). Irritation thus functioned not only as an emotional reaction, but as an affective signal that prompted the residents to reflect ethically on their professional responsibility.

### 4.3 Agency and Power Asymmetries

Appointments with manipulative patients or aggressive relatives showed how irritations arose at the junction of vulnerability and professional boundaries (e.g. Interview 4, Case 1; Interview 9, Case 2). In such moments, irritation marked the point at which the cognitively acknowledged limits of one's professional agency were experienced in an acute and physically perceptible way.

### 4.4 Structural and Organisational Conditions

Problems such as time pressure, lack of documentation, language barriers or disordered home conditions often led to irritation, as they disrupted daily clinical routines (e.g. Interview 11, Case 1; Interview 13, Case 1). These irritations demonstrated how organisational and systemic limitations shape the experience and visibility of uncertainty in everyday life.

## 5 Discussion

Existing studies on medical uncertainty have mainly conceptualised it as a cognitive or epistemic problem [1, 2], and more recently as a practical challenge of clinical work [3, 5]. Within this literature, affective dimensions have often been treated as secondary—as individual reactions to gaps in knowledge or as signals that a diagnostic intuition may be warranted [8]. Our analysis suggests a different starting point: we propose to understand irritation as an affective marker through which residents become aware that something in the consultation is not ‘running as it should’. While gut feeling typically points toward a possibly hidden diagnosis, irritation more often signals that something is wrong in the interaction or workflow—and it is this disruption, not the clinical ambiguity, that leads to uncertainty.

These disruptions of the taken‐for‐granted order can be situated more precisely against existing work along the two axes that organised our results, the interactional and the structural. On the interactional side, Balint [18] already described physicians' irritation and discomfort as meaningful, if largely implicit, signals, yet he located them mainly within the psychodynamics of the doctor–patient dyad; our material suggests that irritation arises not only relationally but also from the interaction order itself and from organisational conditions. The encounters marked by manipulation or aggression (Interview 4, Case 1; Interview 9, Case 2) can thus be read as concrete instances of the conversational ‘dysfunctions’ that Heritage [20] described, drawing on Goffman's account of the interaction order [19]. They also extend this account in two respects: they show how such dysfunctions are first registered affectively, as irritation, before they can be cognitively framed, and they foreground a shift in role and power asymmetry—most strikingly where a patient claims interpretive authority over the physician's own affect. Where irritation instead opened into a deeper grasp of the patient's situation (Interview 12, Case 2), our findings resonate with the ‘heart‐sink’ literature [30] and with anthropological work on illness narratives and explanatory models [15, 16], but add a developmental reading in which an initial affective burden can become a starting point for psychosocial understanding rather than only a strain to be managed.

On the structural side, our analysis connects most directly to accounts that conceive of uncertainty as enacted rather than purely epistemic. Timmermans and Angell [5] distinguished epistemic from practical uncertainty and argued that medical doubt is performed in social and organisational settings; the irritation our residents described under time pressure, language barriers, missing documentation or chaotic home conditions (Interview 11, Case 1; Interview 13, Case 1; Interview 10, Case 1) offers an affect‐centred, empirical illustration of such practical uncertainty. It also marks the point at which residents begin to deploy the more relationship‐oriented coping strategies catalogued by Han et al. [3]—collegial consultation, shared responsibility, improvisation and the use of local networks—by naming the affective trigger that precedes them. At the same time, this is the dimension on which prior research offers the fewest direct points of comparison: studies of affective markers have largely revolved around the intuition–evidence axis [8, 11, 27, 28], leaving the interactional and especially the structural production of uncertainty comparatively under‐theorised. Rather than forcing such comparisons, we read our contribution here as an extension of uncertainty research into this less charted terrain.

Viewing uncertainty through the prism of irritation shifts analytic focus in at least three ways. First, it highlights the temporal development of uncertainty. Irritation presents itself as an initial, bodily felt disturbance, which can only later be articulated as a starting point for doubt, (self‐)reflection or learning. Second, it highlights the ethical and political implications of dealing with uncertainty, as residents weigh risks, time pressure and responsibilities under conditions that are shaped more by institutional logics than by clinical thinking alone. Third, it shows that the awareness of one's own limited capacity to act and the recognition of potentially changing power asymmetries are not accidental. They are the key to understanding how uncertainty is experienced emotionally and how clinicians respond to it in everyday practice.

Our results also underline the ambivalence of irritation. On the one hand, moments of irritation can open trajectories towards deeper understanding of patients: they prompt residents to pause, to reconsider taken‐for‐granted assumptions, and to pay more attention to psychosocial contexts and relational dynamics. On the other hand, irritation may guide attention to the structural limits of health care and highlight what cannot be resolved within the limits of time, resources or institutional rules. These patterns are not mutually exclusive, but they show that uncertainty is not simply a deficit to be overcome. Instead, it is produced and shaped through the interplay of clinical, interactional, and organisational factors and constitutes a routine component of medical decision‐making. Recognising this ambivalence helps to avoid individualising uncertainty as a personal weakness and instead frames it as a structural feature of contemporary primary care.

Finally, conceptualising irritation in this way has implications for residency training and for organisations. If irritation marks the moments when routines break down and uncertainty becomes easy to sense, then these moments are meaningful opportunities for the professional development of residents. However, they become more productive if they can be named, shared and reflected upon. This underlines the importance of institutionalised spaces in which residents and senior physicians can discuss irritations without fear of blame, and of training formats that include communication and de‐escalation in conflict‐laden encounters as well as an explicit focus on organisational and systemic limits. Instead of trying to eliminate uncertainty, such arrangements could help to cultivate a professional stance in which uncertainty and irritation are acknowledged, made discussable and used as resources for professional awareness and learning.

## 6 Limitations

This study is based on a small sample of 15 residents in one German region, which limits the generalisability of the results. Most of the interviews were conducted during the COVID‐19 pandemic, often via video conference, which may have affected the depth of the narrative. The study relied on retrospective self‐reports, shaped by memory or self‐presentation. Furthermore, the described affective markers may be subject to retrospective reinterpretation: irritation may be perceived more clearly in retrospect and may not have been present to the same extent at the time of the encounter. Cases that caused irritation but were not followed up may be remembered less clearly and therefore appear less frequently in the narratives – especially when they ended without a clear or memorable outcome. The restrictions are also of cultural origin: Although the study was open to both native German speakers and people with a migration background, only native speakers participated, so uncertainties related to intercultural encounters may be underrepresented. Cultural factors may also shape how irritation is expressed and interpreted in clinical practice, which limits the generalisability of our findings beyond the German context. Finally, the interdisciplinary background of the research team broadened perspectives but also risked selective emphasis.

## 7 Conclusion

This article has shown how moments of irritation offer an affective approach to medical uncertainty that differs from the widely discussed ‘gut feeling’. Irritation does not point to hidden diagnoses, but arises when communicative or organisational expectations are not met, thus opening a separate path into the experience of uncertainty. In this sense, irritation marks the points at which routines break down and the interrelation of clinical, interpersonal and systemic challenges becomes visible.

For the training of resident physicians, this means that protected spaces are needed where such moments can be discussed, that communication and de‐escalation skills are required to manage disruptive encounters, and that the knowledge of resident physicians about organisational and systemic constraints must be strengthened. As one resident put it: ‘We are all uncertain, even the old hands at 60 are still uncertain. And uncertainty is not dangerous, it is something you can deal with once you start talking about it’ (Interview 5). Expressing irritation and uncertainty does not eliminate them, but it can transform them into resources for vigilance, reflection and professional development, rather than viewing them merely as destabilising experiences.

## Ethics Statement

The study was approved by the institutional review board of the University of Marburg, Germany (Az. Studie 94/19). All participants provided written informed consent prior to participation.

## Conflicts of Interest

The authors declare no conflicts of interest.

## Data Availability

The qualitative interview data are not publicly available. Although the interviews were pseudonymised, the dataset contains detailed case narratives and contextual information that could potentially allow participants to be identified, particularly given the regional and professional context of the study. In addition, participants were informed that the interview recordings would be deleted after completion of the study. Therefore, the data cannot be shared publicly. Selected pseudonymised excerpts are included in the manuscript.
